# Design, Development, and Evaluation of Multimodal Conversational Agents for Health Data Registration and Monitoring: Framework Proposal and Pilot Exploratory Study

**DOI:** 10.3390/healthcare14121641

**Published:** 2026-06-10

**Authors:** Mateus Klein Roman, Luan Zanatta, Jeangrei Emanoelli Veiga, Ericles Andrei Bellei, Ana Carolina Bertoletti De Marchi

**Affiliations:** 1 Institute of Technology, University of Passo Fundo (UPF), Passo Fundo 99052-900, RS, Brazil; 2 Institute of Health, University of Passo Fundo (UPF), Passo Fundo 99052-900, RS, Brazil

**Keywords:** chatbots, personal health record, electronic health records, user experience, conversational agents, voice recognition software

## Abstract

**Objectives:** This study proposes an implementation-oriented design framework for multimodal conversational agents handling patient-generated health data and reports an exploratory experiment evaluating its instantiation in hypertension self-monitoring, focusing on user experience of conversational data-entry workflows. **Methods:** The framework operationalizes four complementary dimensions (social intelligence, communication style, anthropomorphic characteristics, and technological mapping) and was instantiated in two agents integrated into an eHealth platform. Each agent supports users by providing prompts, interpreting responses, checking data plausibility, and confirming submission. A three-arm, single-session feasibility experiment (n=18, n=6 per group) compared a conventional app interface with text-based and voice-based conversational agents. Evaluation triangulated three sources of evidence: open-ended qualitative responses analyzed through descriptive content analysis, session-level researcher observation notes, and the User Experience Questionnaire (UEQ) reported descriptively with one-way ANOVA and $\eta^{2}$ effect sizes. **Results:** All three modalities were acceptable to participants and produced UEQ scores in the positive range. Hesitation was observed in 2 of 6 Control participants, 1 of 6 Text participants, and 3 of 6 Voice participants, with self-reports indicating that voice-related difficulties were modality-specific (diction, command phrasing) and resolved within the session. Qualitative themes of acceptability and innovation, perceived effort, and modality-specific facilitators emerged across the corpus. Between-group ANOVAs did not reach statistical significance (p>0.05), as expected for an underpowered design, yet $\eta^{2}$ values were medium for Attractiveness, Efficiency, Dependability, and Pragmatic Quality and large for Stimulation and Hedonic Quality, converging with the qualitative innovation and engagement signal in the conversational conditions. **Conclusions:** The framework and feasibility experiment provide preliminary, hypothesis-generating evidence on the potential of multimodal conversational interfaces in healthcare. However, no clinical, behavioral, or longitudinal outcomes were assessed. The four design dimensions can be tentatively associated with themes recognizable in user discourse, and the observed effect-size pattern motivates adequately powered longitudinal studies that incorporate behavioral and clinical endpoints alongside user experience measures.

## 1. Introduction

The expansion of digital health technologies has transformed healthcare delivery, enabling remote information exchange and condition management for patients and providers [1]. As health data becomes increasingly integrated into daily routines, individuals depend on robust platforms that support accurate data entry, real-time monitoring, and efficient communication. Traditional methods of health data registration and tracking, however, often introduce obstacles such as complex user interfaces and burdensome data entry requirements, which may discourage consistent patient engagement [2]. Consequently, there is a need for strategies that streamline user interaction, minimize cognitive load, and establish more intuitive user interfaces [3].

In arterial hypertension care, interaction barriers can threaten more than usability by affecting continuity and the clinical utility of self-monitoring [4,5]. If patients record home blood pressure readings inconsistently or inaccurately, care teams may lack sufficient information to adjust treatment, identify uncontrolled patterns, or provide timely self-management guidance [6]. Strategies such as plausibility checks and confirmation of critical values can reduce data entry burden while maintaining quality. These methods help patients adhere to self-monitoring [7,8], generate more reliable records, and support safer, more informed decisions based on patient-generated health data [9].

To address these challenges, recent initiatives have incorporated user-centered design principles to better align digital health tools with users’ needs, expectations, and communication preferences [10]. One promising approach involves integrating multimodal conversational agents, including text-based chatbots and voice-based virtual assistants, into health informatics systems [11,12]. These agents, which emulate natural human conversation, can streamline information recording and retrieval, thereby reducing user effort and minimizing errors in data capture [13]. Furthermore, conversational agents offer opportunities for more engaging and human-like interactions, which may promote long-term adherence and improve overall satisfaction with digital health solutions [14].

Despite the growth of conversational agents, their design and implementation often remain *ad hoc* and lack a clear theoretical framework. Prior research [15,16] identifies four key dimensions for developing multimodal conversational agents in health: social intelligence, communication style, anthropomorphic characteristics, and technological mapping. Social intelligence enables agents to interpret and respond empathetically to user cues, such as emotional tone and context. Communication style refers to adapting language and interactions to users’ health literacy. Anthropomorphic features, including voice and persona, can make agents seem warmer and more trustworthy. Technological mapping involves selecting platforms, data structures, and integration tools that deliver a seamless, interoperable solution.

Although key principles for developing multimodal conversational agents have been identified, a comprehensive model that systematically organizes and applies them remains underexplored [17]. Addressing this gap could advance the field by offering a unified conceptual framework that guides the practical development of conversational agents [18]. Integrating these fundamental design dimensions can support the consistent translation of theoretical constructs into practical solutions, thereby enabling more effective, user-friendly, and interoperable platforms for health data registration and monitoring. This paper presents an exploratory design-and-implementation study that unifies these four dimensions and demonstrates their applicability through the development and experimental evaluation of both voice-based and text-based agents for health data management. It assesses the feasibility and user experience of these agents, focusing on usability and engagement in hypertension self-monitoring data entry, and identifies implementation factors and evaluation endpoints for future real-world deployment. The contribution of this work is therefore threefold: a conceptual contribution in the form of an implementation-oriented framework that organizes the four design dimensions into actionable design decisions; a technical contribution in the form of two working agents, text-based and voice-based, integrated with an operational eHealth platform; and an empirical contribution in the form of an exploratory, single-session user experience evaluation that anchors the framework to user-reported signals while remaining preliminary in scope.

## 2. Methods

The study comprised three stages: framework formulation, development of the conversational agents, and subsequent user experience (UX) assessment.

### 2.1. Design Framework for Multimodal Conversational Agents Handling Healthcare Data

A design framework was established to facilitate the development of multimodal conversational agents for health data entry and monitoring. The framework formulation was guided by two systematic reviews [15,16] that synthesize the existing evidence on conversational-agent design in health, as well as by complementary primary studies that operationalize the constructs at the dimensional level. Each of the four dimensions is anchored in a distinct theoretical tradition: social intelligence draws on relational communication and behavior-change theory [19,20]; communication style, including vocal features, draws on conversational-design and communication-competence research [21,22]; anthropomorphic characteristics draw on social-presence and trust-formation theory in human–machine interaction [23,24]; and technological mapping draws on health-informatics interoperability and middleware-orchestration practice [17,18]. The resulting framework consists of these four dimensions, which are described below. By combining them and their operational characteristics into a single design structure, the framework addresses a scientific gap between isolated design recommendations and implementation-oriented guidance, constituting a key contribution of this study.

#### 2.1.1. Conversational Agent’s Social Intelligence

This dimension includes the essential elements required to accurately interpret user characteristics, with a focus on authenticity, clarity, and empathy. Its primary objective is to prevent erroneous interaction flows by enabling the agent to recognize and adapt to user needs while collecting data accurately. In this study, we posit that chatbots designed for sensitive health topics, when equipped with social intelligence, benefit from establishing a private, nonjudgmental environment. This environment is further enhanced by the use of culturally relevant language and personalized responses, thereby fostering user engagement and openness [19]. Effective health chatbots incorporate behavior-change strategies such as goal setting, real-time feedback, and on-demand support while maintaining the relational capacity needed to build trust and rapport with users [20]. Examples of features derived from this dimension include multimodal blood pressure input options and a conversational grounding loop (uptake, plausibility validation, and explicit read-back confirmation, with repair prompts for missing or invalid values).

#### 2.1.2. Communication Style and Vocal Features

This dimension addresses the tailoring of the agent’s vocal attributes and communication patterns to match the preferences and expectations of the target population, namely patients undergoing treatment and health monitoring. Selecting communication styles and vocal qualities that align with user expectations increases familiarity and fosters more engaging interactions. In our design approach, we draw on evidence from the literature regarding the use of personal pronouns, empathetic and respectful responses, strategic wording, and a three-stage conversational framework that emulates human-like dialogue to enhance naturalness and user comfort [21]. Communication-competence strategies such as empathetic responses, contingency, humor, small talk, emotional expressiveness, personalization, social etiquette, and explanation can promote more positive user perceptions of conversational agents [22].

#### 2.1.3. Anthropomorphic Characteristics

Integrating human-like features into the agent increases user engagement and acceptance. These characteristics help bridge the gap between human and machine communication, resulting in a more immersive, user-centered environment for health data interaction. In the health domain, chatbot personality traits, such as warmth and competence, as well as perceived gender and role (e.g., prevention or therapy), influence perceived credibility, user satisfaction, and ultimately the intention to use the chatbot [23]. Anthropomorphic features enhance users’ perceived social presence, communication quality, and trust, which, in turn, increase intentions to reuse the technology [24]. In this study, we employed characteristics that enhance anthropomorphism, such as more natural, fluid, and non-rigid language. For instance, we refrained from using numerical options that resemble menu navigation, avoided a rigid input–output style, avoided the imperative mood, customized memory based on previously mentioned user inputs, ensured variation in responses to avoid uniformity, and incorporated markers of orality. The deliberate selection of Alexa as the platform was motivated by its inherent naturalness and its ability to support customizations that seamlessly integrate these features.

#### 2.1.4. Technological Mapping

This dimension involves systematically identifying and aligning the technologies required to implement conversational agents in an eHealth context. It encompasses selecting tools and platforms that facilitate data input, processing, interoperability, and integration with existing health information systems. Effective technological mapping supports the seamless and efficient deployment of conversational agents for health data management. At an operational level, this mapping is structured as a stepwise inventory of all components that must interact within an eHealth environment. Specifically, it involves: (i) defining the conversational front-end(s) and establishing how their intent and slot schemas connect to the clinical data model; (ii) outlining the integration layer responsible for translating conversational payloads into platform actions; and (iii) detailing data governance mechanisms that ensure privacy, security, auditability, and the proper management of clinically relevant values. This operational decomposition clarifies how each subsystem contributes to the overall interoperability and safe operation of the agents.

### 2.2. Development of the Conversational Agents

The conversational-agent framework was integrated into the existing eHealth platform, eProHealth, to support both text and voice modalities, based on four key design dimensions. The platform includes features to support data safety and privacy. It enables the monitoring of patients with hypertension and supports risk assessment, behavior change guidance, alerts, reminders, and healthcare support. Users manually enter several health indicators, such as blood pressure, heart rate, body temperature, blood oxygen saturation, weight, waist circumference, body fat, physical activity, sleep, mood, and lipid levels.

The text-based solution was developed using Amazon Lex, a tool for building conversational interfaces that leverages Amazon Alexa technology. These platforms were selected for their ease of integration, broad adoption, and customization features. They support natural-language interactions with anthropomorphic qualities and are compatible with future integration of Amazon Polly voices.

In preparing the solution for the Amazon Alexa app and other conversational devices, technological feasibility and the backend infrastructure required to manage external data were carefully evaluated. The existing platform server was used to provide data access and support record insertion, modification, and deletion. Development and initial testing occurred locally, followed by integration into the Amazon cloud environment to ensure compatibility with both Amazon Lex and Amazon Alexa. From an implementation standpoint, both conversational front-ends relied on the existing eProHealth server to execute business rules and persist health records.

The server-side component follows a Java-based Model–View–Controller architecture, exchanges JSON payloads, and secures requests using JWT authentication. For the text-based agent, Amazon Lex invokes an AWS Lambda function that receives slot values, validates and formats them, and sends REST requests to the eProHealth API to create, retrieve, and delete records; it then returns the operation status to the user. For the Alexa skill, three backend strategies were considered: two Alexa-hosted options (Node.js or Python 3.12) and an external endpoint. We selected the external eProHealth server to reuse the existing API and centralize governance and business logic. All system functionalities and specifications were implemented in accordance with the required privacy, security, regulatory safeguards, and authorization procedures.

The three conditions differ along a few technical dimensions worth summarizing. The conventional app is the platform’s existing graphical interface, where users enter values through standard form controls and on-screen navigation. The text-based chatbot, built on Amazon Lex, replaces form filling with typed natural-language turns: utterances are mapped to intents, numeric values are captured through custom slots, entries are checked against plausibility ranges, and a bounded retry policy governs correction. The voice-based agent, built on Amazon Alexa, uses the same intent-and-slot logic over spoken input, and introduces an explicit read-back-and-confirm step before a value is persisted. All three write to the same eProHealth records through the platform server, so the conditions differ in the interaction surface and the input modality rather than in the underlying data model or storage. This shared backend is what supports a like-for-like user-experience comparison across modalities.

### 2.3. Evaluation of User Experience

The experiment was conducted on-site at public healthcare facilities in Carazinho, Rio Grande do Sul, Brazil, with ethical approval from the local Research Ethics Committee (opinion number 5391765). The study population comprised men and women aged 40 to 69 years with a prior diagnosis of hypertension who provided informed consent. Eighteen participants were selected using non-probabilistic convenience sampling. The minimum age criterion of 40 years was based on literature recommendations and corresponded to the average age of chronic disease onset in Brazil [25].

Participants were allocated to three groups using block randomization. Allocation occurred sequentially as participants were recruited, following a pre-generated randomization list, and the upcoming assignment was concealed until a participant had been enrolled, so recruitment was not influenced by knowledge of the next group. The first group used a voice-based conversational agent (Voice). The second group used a text-based agent (Text). The third group (Control) used the platform’s conventional app. Data collection took place in a single session and followed a standardized task script (insertion, retrieval, and deletion of health records); the scripts for each group are available in the Supplementary Files. First, participants completed a demographic questionnaire covering age, education, income, and technology experience, including a specific item on prior use of voice assistants that named Alexa and Google Home as examples. Next, they interacted with the assigned solution for about 15–30 min.

While each participant interacted with the assigned solution, the researcher conducting the session recorded a session-level observation note indicating whether the participant exhibited hesitation or difficulty during task execution. For this note, hesitation or difficulty was counted when a participant paused visibly before acting, asked the facilitator for help or clarification, repeated or retried a step, or voiced uncertainty about how to proceed. The note used a binary observed/not observed criterion at the session level and was recorded by a single observer who applied this fixed criterion alongside the participant’s own self-report. The observer was not aware of the study’s directional hypotheses, although, as the person facilitating the session, the observer was necessarily aware of the assigned modality. We treat this note as an informal, complementary field observation rather than as a validated or objective performance measure, and it complements the qualitative analysis and the UEQ instead of supporting comparative conclusions between groups. Finally, participants completed the User Experience Questionnaire (UEQ) [26], administered in its validated Portuguese version and later scored with the official UEQ analysis tool following the instrument’s standard procedure, and an open-ended questionnaire for qualitative feedback (available in the Supplementary Files), with optional fields for describing overall experience, perceived difficulties, perceived facilitators, and suggestions for improvement.

Given the exploratory and underpowered nature of the design, the analysis plan emphasized triangulation across qualitative themes, observational signals, and descriptive UEQ measures, with inferential testing reported as hypothesis-generating rather than confirmatory. Quantitative data were analyzed using the proprietary UEQ analysis tool [26] and the Jamovi 2.0 statistical package [27]. Per-scale means, standard deviations, and 95% confidence intervals were reported for each group. One-way ANOVA tests were used to compare groups across UEQ scales using a 5% significance threshold (p<0.05); these comparisons were exploratory and descriptive and were not designed to establish superiority between modalities. Given the small sample size, eta-squared ($\eta^{2}$) effect sizes were also computed and interpreted according to Cohen’s benchmarks ($\eta^{2}\;\approx \;0.01$ small, 0.06 medium, 0.14 large) [28]. Because effect-size estimates are imprecise at this sample size, the magnitude labels were treated as descriptive aids for interpretation and hypothesis generation rather than as stable estimates. The effect sizes supported interpretation in the absence of statistical significance and provided inputs for sample-size planning in future confirmatory studies. The session-level observation notes were summarized as the count and proportion of participants in each group for whom hesitation or difficulty was flagged, alongside the participants’ own descriptions in the open-ended responses.

Open-ended responses were analyzed using a descriptive content-analysis approach [29]. All responses provided by participants were included in the analysis. Coding was inductive: the categories were derived from the responses themselves rather than from a predefined scheme, and they were organized around the four elicitation prompts of the open-ended questionnaire (overall experience, perceived difficulties, perceived facilitators, and suggestions for improvement). A single coder coded the full corpus and grouped the codes into recurrent themes, which were then compared across groups; representative excerpts were retained to illustrate each theme. A second researcher reviewed the original responses and the resulting codification as a verification step. Given the small corpus, the qualitative findings are presented as illustrative rather than as evidence of thematic saturation. Because data collection took place in Brazil with native Portuguese-speaking participants, all verbatim responses were originally recorded in Brazilian Portuguese. For presentation in this manuscript, illustrative excerpts were translated into English by the authors with the aim of preserving meaning while smoothing typographical irregularities present in the originals. To ensure comparability across conditions, standardized task scripts were used for the text-based agent, voice-based agent, and conventional app sessions. The scripts are available in Supplementary Files.

We distinguish several related constructs that are sometimes used interchangeably. Usability refers to how effectively, efficiently, and comfortably a user can complete tasks with an interface. UX is broader and spans the pragmatic and hedonic qualities of the interaction; it is what the UEQ measures [26]. Engagement refers to sustained, repeated use over time; acceptability refers to whether users find a solution appropriate and are willing to use it; and adoption refers to actual uptake in routine practice. This study measured UX and elicited acceptability-related impressions through open-ended feedback. It did not measure engagement, adoption, or adherence, which require longitudinal data. We therefore read positive UEQ scores as evidence of a favorable UX, and any reference to engagement is an interpretive hypothesis rather than a measured outcome.

## 3. Results

The interaction flow for both the text- and voice-based solutions included the retrieval, deletion, and addition of five categories of health records: blood pressure, heart rate, sleep, body fat, and weight. The text-based chatbot received the official software registration number BR5120240012460 in Brazil. The voice-based agent received registration number BR5120240012478.

### 3.1. Text-Based Conversational Agent

The text-based conversational agent was developed exclusively on the Amazon Lex platform, leveraging Lex’s built-in natural-language-processing (NLP) and visual workflow tools. Using the platform’s visual interface, the required intents, entities, and contexts were designed. To support task-oriented interactions, intents were organized into three functional clusters: (i) data collection (adding records), (ii) record retrieval, and (iii) record deletion. For each health indicator, training utterances supported both single-turn multi-slot capture and guided step-by-step completion.

During development and evaluation, the agent was accessed via a web chat interface provisioned with the Amazon Lex Web UI template and deployed via AWS CloudFormation, generating a link that could be embedded in external websites or applications. Representative training utterances for the intent of entering blood pressure records, provided in Portuguese, included: “*Pressure*; *Dictate pressure*; *Add pressure*; *Register pressure*; *Insert* {systolic value} *by* {diastolic value} *of blood pressure*; *Insert* {systolic value} *by* {diastolic value} *of BP*; *Add* {systolic value} *by* {diastolic value} *of blood pressure*; *Add* {systolic value} *by* {diastolic value}”.

The platform includes entities, called slots, within training utterances to capture workflow-specific information. In this context, the {systolic value} and {diastolic value} fields are custom numeric slots designed to record blood pressure values. For example, users can enter “*Add 120 by 80 for blood pressure*” or “*Add 120 systolic and 80 diastolic pressure*,” enabling the system to interpret each measurement automatically. A validation feature detects implausible or reversed values and prompts the user to enter a new value when an entry falls outside the accepted physiological range, defined here as 70 to 250 mmHg for systolic and 30 to 140 mmHg for diastolic pressure. A missing value for a required field triggers a recall prompt, so the record cannot be completed until the field is supplied. Once completed, the data are synchronized with the central database of the digital health platform.

After slot validation, Amazon Lex triggers the AWS Lambda orchestration function. This function translates the slot payload into a platform request and handles the response. To reduce user frustration and avoid infinite correction cycles, the agent uses a bounded retry policy. After three invalid inputs for a required slot, the flow is aborted, and the user is returned to the initial prompt.

### 3.2. Voice-Based Conversational Agent

For the Amazon Alexa voicebot implementation, each intent was designed to ensure that voice input was validated and confirmed. Users can provide multiple slots in a single utterance, allowing the system to process multiple pieces of information at once, similarly to the text-based version. Numeric values (e.g., systolic and diastolic pressure) use the built-in AMAZON.NUMBER slot type. Custom slot values handle variables outside the default model, such as affirmative or negative medication responses (e.g., “*yes*”, “*no*”, or “*I did not take it*”). To reduce speech-recognition errors, the voice agent repeats critical values and asks users to confirm each measurement before submission. This feature is particularly useful for ensuring accuracy when handling health data.

**Figure 1:**
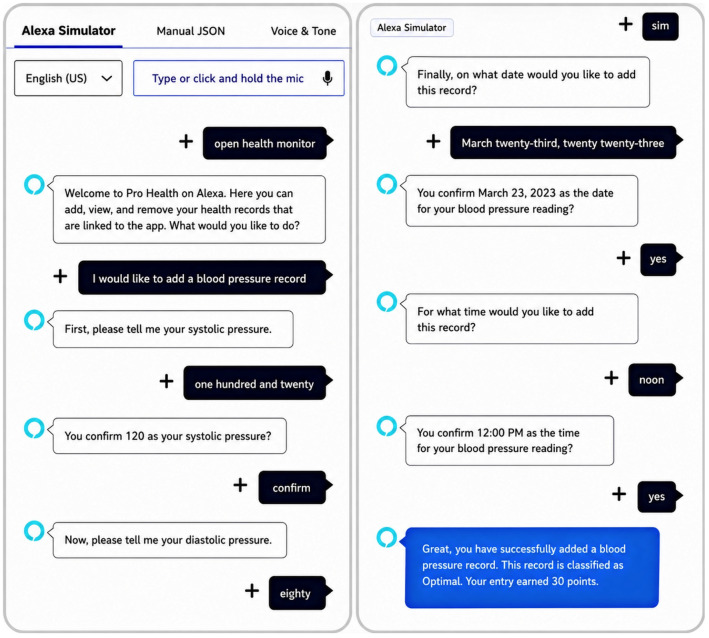
Demonstration of the voicebot in the Alexa conversational simulator. The interface displays a conversation in which a user logs their blood pressure. The dialogue shows the bot guiding the user through step-by-step prompts to collect systolic and diastolic values. The interaction concludes with the system confirming the entry and classifying the reading. This is a translated version of the original screenshots in Brazilian Portuguese.

A validation routine was implemented in the voicebot to verify the accuracy of each required slot. For every mandatory variable needed to fulfill an intent, this routine checks whether the user’s input meets the specified criteria. If, for instance, a user attempts to record a value that does not meet systolic-pressure requirements, the platform automatically rejects the input and prompts for a corrected value, applying the same accepted physiological ranges used by the text-based agent. To further ensure data accuracy, the voicebot repeats the recorded values to the user, providing an opportunity for confirmation or correction before submission. Figure 1 presents the voice-interface simulator screen and its corresponding textual transcriptions.

### 3.3. User Experience Measurements

#### 3.3.1. Sample Characteristics and Baseline Equivalence

Eighteen individuals participated in the evaluation and were assigned to the Text, Voice, and Control groups in equal numbers (n=6 per group). The sociodemographic characteristics of the participants are presented in Table 1. The three groups were broadly comparable on demographic and technology-familiarity variables, including gender, age band, frequency of mobile-device use, prior knowledge of conversational agents, and self-assessed level of technological knowledge. Group-comparison tests yielded p>0.05 for all variables, supporting baseline equivalence and comparability for subsequent analyses. Within the limits of a small convenience sample, no group was advantaged by *a priori* differences in digital experience or familiarity with conversational interfaces.

#### 3.3.2. Session-Level Observational Signals

During each session, the researcher recorded whether the participant exhibited hesitation or difficulty while completing the task script. Hesitation was flagged in 2 of 6 Control participants (∼33%), 1 of 6 Text participants (∼17%), and 3 of 6 Voice participants (50%). These proportions are descriptive only; with six participants per group, they are not treated as a ranking of the modalities. The participants’ own self-reports, captured through the open-ended questionnaire, were consistent with the observer notes: Voice participants who were flagged described early difficulty with diction and with formulating spoken commands (e.g., *“Difficulty with the diction of the phrase”*, *“At first I had a sense of what to do, but I struggled while using the solution to figure out how to speak”*), whereas Control participants who were flagged attributed difficulty to general unfamiliarity with mobile applications rather than to the specific tasks. These observational signals are coarse, single-observer indicators and should be interpreted as descriptive feasibility evidence rather than as formal performance metrics, but they provide a complementary view of perceived effort that does not depend on subjective scale ratings. Objective task performance indicators (for example, task completion rate, task duration, and error or correction counts) were not collected in this single feasibility session, so the hesitation note and the open-ended self-reports are the only effort-related signals available here.

#### 3.3.3. Qualitative Themes from Open-Ended Feedback

Open-ended responses across the four aspects (overall experience, perceived difficulties, perceived facilitators, and suggestions) were synthesized into recurrent themes and compared across groups. Table 2 summarizes the comparative qualitative findings, and the paragraphs below organize them around three cross-cutting themes that emerged from the corpus. Given the small corpus and the use of a single coder, these themes are presented as illustrative patterns rather than as saturated categories.

*Theme 1—Acceptability and perceived innovation:* Participants across all three groups described the experience in positive terms (e.g., *“Very good”*, *“I found it very good”*, *“Interesting for monitoring”*), suggesting broad acceptability of both conversational and conventional data-entry tasks for hypertension self-monitoring. Explicit references to innovation, however, were concentrated in the conversational conditions: Text participants described the experience as *“Innovative”* (e.g., *“It was an innovative experience for current times”*), and Voice participants framed the interaction as *“innovative, new technology”*. Control participants reported a positive experience but more often described the app as *“easy to handle”* or as offering *“a new way of learning about pathologies”*, which suggests familiarity coupled with educational value. The pattern indicates that conversational modalities carry a stronger novelty signal that may favor engagement, while the conventional app benefits from low entry friction.

*Theme 2—Perceived effort and learning curve:* Reported difficulties differed in nature across conditions, beyond differences in frequency. In the Control group, the two participants who reported difficulty attributed it to general unfamiliarity with mobile applications (e.g., *“Because of my difficulty using applications of any kind”*, *“A little, because I do not know how to handle apps”*), pointing to a digital-literacy barrier independent of the task. In the Text group, only one participant reported any difficulty, framing it as a personal lack of experience rather than as friction with the interface (e.g., *“Innovative, but because I lack experience I find things difficult”*). In the Voice group, half of the participants described an initial adaptation cost specific to the modality, including diction problems and uncertainty about command phrasing (e.g., *“Difficulty with the diction of the phrase”*, *“At first my experience was confusing, but as it went along I adapted, and in the end everything was fine”*). This last quote is informative because it captures a pattern in which initial friction resolves within a single short session, suggesting that the learning cost is bounded and may be addressable through onboarding and prompt design rather than indicating a fundamental usability barrier.

*Theme 3—Facilitators and modality-specific strengths:* Each modality surfaced distinct facilitators. Control participants emphasized logical task flow and speed (e.g., *“The flow of each block”*, *“Speed, readable app”*), Text participants emphasized simplicity and ease of comprehension (e.g., *“It was very easy and simple”*, *“Easy to understand”*), and Voice participants emphasized guidance and verifiability (e.g., *“The explanations”*, *“The guidance”*, *“Ease of recording and verifying the data”*). The Voice references to verification mirror the agent’s confirmation-and-read-back mechanism, suggesting that participants perceived the explicit confirmation step as a positive design feature rather than as interaction overhead. Suggestions for improvement converged on simplifying deletion (Control), broader chronic-condition coverage (Control and Text), and continued development for healthcare deployment (Text and Voice), pointing toward longitudinal extensions of the design rather than fundamental redesign.

#### 3.3.4. User Experience Scores of UEQ

**Figure 2:**
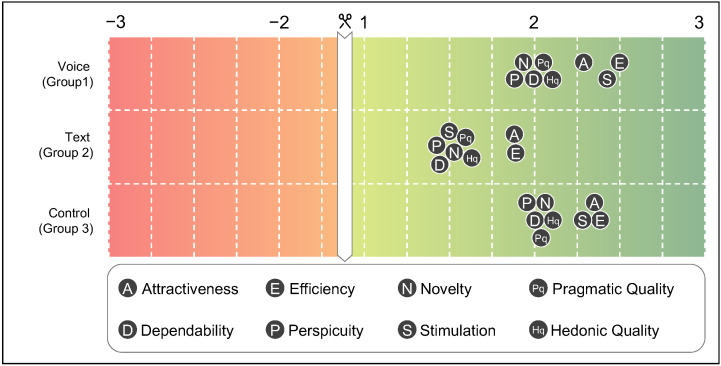
Comparative UX assessment between groups, with mean scores on the UEQ scale ranging from −3 to +3. To read the figure, scores above zero indicate a positive evaluation, and the scales group into pragmatic quality (Perspicuity, Efficiency, Dependability) and hedonic quality (Stimulation, Novelty), with Attractiveness as an overall valence dimension. In this exploratory pilot study (n=6 per group), analysis of variance did not detect statistically significant between-group differences (p>0.05 for all tests). Effect sizes ($\eta^{2}$) ranged from small to large across scales, with the largest magnitudes observed for Stimulation and Hedonic Quality. The per-scale numeric detail (per-group means, SDs, 95% CIs, ANOVA terms, $\eta^{2}$, and magnitude) is reported in full in Table 3.

The descriptive UEQ profile across groups, scales, and the pragmatic and hedonic dimensions is shown in Figure 2, with the full numeric output (per-group means, standard deviations, 95% confidence intervals, ANOVA terms, effect sizes, and Cohen’s-benchmark magnitudes) consolidated in Table 3. All three groups scored in the positive range (>0.8 on the −3 to +3 scale) on every UEQ scale, and all three exceeded the mean of the official UEQ benchmark dataset, the established reference standard distributed with the UEQ analysis tool (468 studies, 21,175 participants). Scores varied across groups within this satisfactory range, yet the wide and overlapping 95% confidence intervals reported in Table 3 show that the design was not powered for comparison, so any between-group differences are presented descriptively and should not be read as evidence that one modality outperformed another.

Between-group ANOVAs did not detect statistically significant differences on any UEQ scale (p>0.05 for all tests). Given the small sample size and the resulting limited statistical power, $\eta^{2}$ effect sizes were computed to inform interpretation and future sample-size planning. As reported in Table 3 alongside the descriptive statistics, the effects were small for Perspicuity and Novelty, medium for Attractiveness, Efficiency, Dependability, and Pragmatic Quality, and large for Stimulation and Hedonic Quality, although none reached conventional significance. Two interpretations follow. First, the magnitude of the hedonic effects is consistent with the qualitative theme of innovation and engagement that emerged predominantly in the conversational conditions. Second, the pattern is hypothesis-generating rather than confirmatory: with n=6 per group, the study was underpowered to detect medium effects at α=0.05, and the present results should be interpreted as feasibility evidence and as inputs for designing adequately powered confirmatory studies.

## 4. Discussion

### 4.1. Principal Findings

This study examined how multimodal conversational agents (a text-based chatbot and a voice-based agent), guided by a four-dimensional design framework, can support health data registration and monitoring. The UX of the text-based, voice-based, and conventional app modalities was compared in a feasibility experiment drawing on three converging sources of evidence: open-ended qualitative responses, session-level observational notes, and descriptive UEQ scores. Read together, these sources suggest that all three interaction modalities were acceptable to participants, with the conversational conditions carrying a stronger novelty and engagement signal and the conventional app benefiting from low entry friction. UEQ scores were consistently positive across scales, the qualitative corpus described the experience as satisfactory across groups, and observational notes flagged hesitation in a minority of sessions, concentrated in the Voice condition. These patterns align with prior work showing that conversational agents can improve perceived experience and accessibility in health-related interactions and may help reduce interface complexity while supporting users with different literacy and ability profiles [30,31,32].

### 4.2. Potential Healthcare and Interaction Implications

From a healthcare perspective, the practical value of these interfaces depends on whether improved interaction translates into more meaningful self-monitoring processes for hypertension management [33]. In this study, we focused on UX as a feasibility signal and did not assess clinical outcomes, so the care-relevant uses discussed here are potential implications rather than demonstrated benefits. The proposed approach is intended to support specific use cases, such as facilitating routine home blood pressure logging, supporting the completeness of longitudinal records for follow-up visits, and reducing the chance of registration errors through validation and confirmation turns [6]. In the text-based agent, custom numeric slots were validated against acceptable ranges, and implausible or reversed blood pressure values triggered correction prompts before submission. In the voice-based agent, required slots were validated and clinically relevant values were repeated and explicitly confirmed before continuing. In both modalities, bounded retries and clarification prompts were used to reduce infinite correction loops, and middleware orchestration centralized business rules before writing records to the health platform. Together, these mechanisms operationalize a safety-oriented interaction design intended to reduce the likelihood of implausible entries [9]. Two boundaries of this design warrant explicit statement. First, because validation rests on physiological range and plausibility, a value that is incorrect yet falls within range and is confirmed by the user can still be stored, so the safeguards reduce rather than eliminate erroneous entries. Second, for voice entry the residual risk of misrecognition is mitigated by the read-back-and-confirm step but is not removed since a misheard value that the user confirms would persist. During the study, the records were visible only to the participant and to the health professional the participant chose to share them with; the research team had no access to the entered values and did not review submission logs. These points are deployment-readiness considerations rather than findings from the present evaluation.

Across modalities, the descriptive UEQ profile of the voice-based agent was favorable on hedonic dimensions (Stimulation and Hedonic Quality), and the qualitative corpus echoed the same signal: Voice participants described the modality as innovative and noted explicit guidance and verifiability as facilitators (e.g., *“The explanations”*, *“The guidance”*, *“Ease of recording and verifying the data”*). These references mirror the agent’s confirmation-and-read-back mechanism, suggesting that the explicit confirmation step was perceived as a positive design feature rather than as interaction overhead. The same Voice condition concentrated the highest observed hesitation rate (3 of 6), with self-reports pointing to diction and command phrasing in the early minutes of the session and explicit mentions of within-session adaptation (e.g., *“At first my experience was confusing, but as it went along I adapted, and in the end everything was fine”*). Read together, these signals are consistent with a profile in which voice interaction increases perceived engagement and accessibility while introducing a bounded short-term learning cost that may be addressed through onboarding and dialogue design [34,35]. This profile is particularly relevant for users who experience difficulties with typing or fine-motor interaction and for older adults, who comprised the present sample [36]. The text-based chatbot produced UEQ scores in the same satisfactory band, with the lowest observed hesitation rate (1 of 6) and qualitative emphasis on simplicity and ease of comprehension. This pattern is consistent with text-based conversational scaffolding offering low entry friction at the cost of less hedonic distinctiveness, even when supported by validation routines and bounded retries. The trade-off reinforces the need for robust error-recovery strategies (e.g., clear clarification prompts and simple correction paths) to avoid premature task abandonment [34].

### 4.3. Interpreting the Quantitative Signals

Inferential testing did not identify statistically significant between-group differences (p>0.05 for all tests), which should be interpreted in light of the pilot design (small sample, between-group variability in subjective measures, and single-session exposure). Effect-size analysis added nuance to this null finding. While effects were small for Perspicuity and Novelty and medium for Attractiveness, Efficiency, Dependability, and Pragmatic Quality, the magnitudes were large for Stimulation and Hedonic Quality. Given the limited statistical power at this sample size, the absence of significance does not by itself imply that the modalities are equivalent on hedonic dimensions. The descriptive pattern, the effect-size pattern, and the qualitative theme of innovation and engagement converge on a hypothesis-generating signal: conversational modalities, voice in particular, may carry hedonic advantages worth testing in adequately powered confirmatory designs. The combination of pragmatic qualities (e.g., efficiency and dependability) with positive hedonic perceptions (e.g., stimulation and novelty) is consistent with evidence that both dimensions shape overall UX judgments [37].

### 4.4. Implementation and Deployment Considerations

From an implementation perspective, the study highlights trade-offs that are central to health data capture. Confirmation turns and slot-level validation in the voice agent can improve reliability for clinically meaningful values, yet they increase the number of steps and may affect perceived efficiency. The middleware-based orchestration used in both agents supports reuse across modalities and consistent integration with the platform’s database. This approach aligns with the technological mapping dimension and reinforces interoperability-oriented development. In terms of healthcare impact, these patterns are compatible with prior findings. Interactive and personalized technologies may strengthen adherence to self-monitoring routines and behavior-change guidance in chronic condition management [38,39,40]. Moreover, the positive perceptions reported here are consistent with the possibility that conversational agents may foster engagement and habit formation over time, a pattern that requires longitudinal confirmation [41].

Real-world deployment also introduces practical challenges that extend beyond interface usability. For voice-based agents in particular, privacy and governance are central because voice interactions may contain sensitive health and contextual information. Practical safeguards include explicit informed consent, clear disclosure of data flows and retention, encryption, and strict access control [42]. In parallel, adoption depends on equitable access to compatible devices, as well as on users’ digital literacy and language familiarity. Therefore, implementation strategies may include multimodal alternatives, progressive onboarding, and fallback paths when speech recognition or connectivity fails. These considerations are essential for translating pilot UX findings into scalable and responsible routine-care deployment. The favorable UX reported here should also be read against the study’s guided design: standardized task scripts reduced task failure and likely inflated perceived ease of use relative to free, unguided interaction, an effect most consequential for the voice agent, where formulating spoken commands without guidance is itself a usability challenge. Future research should expand the evaluation to encompass larger sample sizes and adopt longitudinal designs to assess sustained engagement and adherence, especially beyond the initial novelty effect [4]. Objective metrics, such as task duration, completion rate, and data-entry errors, can be used alongside self-reported UX to reveal additional patterns.

Data governance in this study followed the Brazilian General Data Protection Law (LGPD) and the protocol approved by the Research Ethics Committee. Processing relied on Amazon Web Services in the South America (São Paulo) region, and health data were transmitted and stored using authenticated, encrypted exchange protocols between the agents and the eProHealth API. Participants were informed of third-party cloud processing through the terms of use, the privacy policy, and the informed consent they signed. We also state plainly the limits of developer and researcher control: voice interactions are processed by Amazon Lex and Alexa, which may retain interaction logs internally under the platform’s own terms, a layer that sits outside the research team’s control. Within that constraint, we applied the storage, handling, and access control measures that the LGPD requires for identifiable health data. The records persisted by the platform fall under the platform’s governance, whereas the voice layer is handled by Amazon under its own terms; sustained deployment should therefore rest on an explicit data-processing agreement that fixes retention periods, deletion rights, and audit access across both layers.

### 4.5. Connecting the Design Framework to the Qualitative Findings

The four-dimensional design framework was operationalized into concrete agent features prior to evaluation. The qualitative corpus from the feasibility study now offers a first, preliminary illustration for each dimension, helping to clarify how theory-derived design decisions translated into perceptions reported by older adults engaged in hypertension self-monitoring. The mapping below is exploratory and bounded by the pilot sample, but it illustrates how each dimension can be tied to a recognizable signal in user discourse, which, in turn, provides a more theory-driven articulation of the framework than the design rationale alone. Because the study measured UX rather than the four dimensions as latent constructs, each association below is one plausible interpretation among others, and some signals may reflect general usability as much as the specific dimension to which they are mapped.

*Social intelligence* was operationalized through plausibility validation, repair prompts for missing or invalid values, and an explicit read-back-and-confirm loop on clinically relevant fields [19,20]. The qualitative theme of perceived guidance and verifiability, expressed by Voice participants as *“The explanations”*, *“The guidance”*, and *“Ease of recording and verifying the data”*, maps onto this dimension and indicates that the agent’s grounding mechanisms were noticed and valued by participants rather than experienced as friction. Perceived guidance and verifiability may also reflect general usability and clear interaction design rather than social intelligence specifically, so we read this association as suggestive rather than as direct evidence for the construct.

*Vocal features and communication style* was operationalized through personal pronouns, empathetic and respectful phrasing, and a three-stage conversation pattern intended to emulate human-like dialogue [21,22]. The qualitative theme of perceived effort and learning curve maps onto this dimension. Reports of initial diction difficulty followed by within-session adaptation (e.g., *“as it went along I adapted, and in the end everything was fine”*) suggest that the chosen communication style was learnable in a single short session for this population, while also pointing to dialogue design refinements (e.g., onboarding utterances and recovery prompts) that could shorten the adaptation phase further.

*Anthropomorphic characteristics* were operationalized through naturalistic phrasing, avoidance of menu-like numerical options, response variability, and orality markers, with the deliberate selection of Alexa as a platform that supports those choices [23,24]. The qualitative theme of acceptability and perceived innovation, expressed across both conversational conditions through repeated use of *“Innovative”* and *“new technology”*, maps onto this dimension. These references need not indicate perceived anthropomorphism since they may express novelty or unfamiliarity with the modality, so we treat the link to anthropomorphic design as interpretive rather than demonstrated. Read with that caution, the pattern is consistent with the idea that anthropomorphic cues may contribute to a sense of novelty and warmth that can be translated into engagement.

*Technological mapping* was operationalized as a stepwise inventory of front-ends, integration layers, and data-governance mechanisms (Lex/Alexa front-ends, Lambda middleware, the eProHealth REST API, JWT-secured exchanges, and bounded retry policies). The qualitative theme of facilitators and modality-specific strengths reflects this dimension at the interaction surface: Control participants emphasized logical task flow and speed (*“The flow of each block”*, *“Speed”*), Text participants emphasized simplicity and ease of comprehension, and Voice participants emphasized verifiability. Each strength can be traced to specific architectural choices (block-structured intents, slot validation, and read-back confirmation), reinforcing the technological mapping dimension as the layer through which the other three become observable to the user.

## 5. Limitations, Learnings, and Implications for Future Research

This study has limitations that should guide interpretation. The sample was small (n=18), recruited by convenience, and evaluated in a single experimental session, which limits broader generalizability and statistical power. With n=6 per group, the design was underpowered to detect medium effects at α=0.05, so the absence of statistically significant between-group differences should be interpreted as expected for a feasibility experiment. The reported effect sizes nonetheless inform interpretation and provide inputs for sample size planning in confirmatory follow-up studies.

The qualitative analysis relied on a single coder, with a second researcher verifying the codification but without a formal inter-rater reliability computation, so the thematic interpretation carries an inherent degree of subjectivity. The session-level hesitation note was likewise recorded by a single observer using a binary criterion and without inter-rater reliability, which is suggested as a practice for future studies. The experiment also did not collect hard objective task performance metrics such as task completion rate, task duration, error and correction counts, data entry accuracy, and abandonment rate; the hesitation note is a coarse complement to these measures, and they are named as endpoints for an adequately powered follow-up.

The guided, scripted nature of the tasks are an inherent limit of user studies since participants were not freely exploring the system in a naturalistic home-monitoring context. Because each participant interacted with the assigned modality only once, a novelty effect may also be present, so the favorable hedonic perceptions could partly reflect initial unfamiliarity rather than a durable preference. The healthcare-relevant constructs evaluated here primarily pertained to UX and acceptability, factors that the literature suggests may facilitate improved health outcomes. However, behavioral outcomes (e.g., adherence to home blood pressure logging), longitudinal engagement, and clinical indicators were not assessed and are explicitly identified as endpoints for follow-up research. The results should therefore be interpreted primarily as exploratory evidence of feasibility and as hypothesis-generating user experience patterns, encouraging further confirmation in larger, longitudinal studies.

## 6. Conclusions

This study adopted a design-and-implementation approach to multimodal conversational agents for the registration and monitoring of patient-generated health data and evaluated its feasibility in hypertension self-monitoring through two instantiations (a text-based chatbot and a voice-based agent) integrated into an eHealth platform. The evaluation was framed as an exploratory feasibility experiment and triangulated qualitative themes from open-ended feedback, session-level observational notes, and descriptive UEQ scores. All three modalities were acceptable to participants and yielded UEQ scores in the positive range. The conversational conditions showed a stronger signal of perceived innovation and engagement, whereas the conventional app benefited from low entry friction. Between-group differences did not reach statistical significance, as expected for an underpowered design.

The primary contribution of this work is an implementation-oriented framework. It links four actionable design dimensions (social intelligence, communication style, anthropomorphic characteristics, and technological mapping) to concrete architectural and dialogue decisions. These include intent/slot modeling, validation routines, confirmation strategies, error handling, and middleware-based integration with the health platform. The qualitative corpus from the feasibility study provided a preliminary and interpretive anchor for each dimension, with grounding mechanisms associated with perceived guidance and verifiability, communication style associated with a learning curve that resolved within a single session, anthropomorphic features tentatively associated with references to innovation and novelty, and technological mapping associated with modality-specific facilitators. This mapping provides a reusable basis for designing and evaluating interoperable conversational solutions in digital health. The framework is therefore the primary and most transferable contribution of this work, while the empirical evaluation remains preliminary and feasibility-oriented.

The patterns observed in this exploratory study should be interpreted as hypothesis-generating rather than confirmatory. They suggest several directions for follow-up confirmatory research: validation and confirmation mechanisms may improve perceived dependability and potentially reduce data-entry errors; conversational modalities may offer hedonic advantages over a conventional interface; and voice interaction may increase engagement at the cost of a bounded short-term learning phase. Future research should test these hypotheses with adequately powered samples and longitudinal real-world deployments, incorporating objective performance and data quality indicators (e.g., task time, error rate, and record completeness), behavioral and clinical endpoints relevant to hypertension self-monitoring (e.g., adherence to home blood pressure logging), and adaptive dialogue strategies that balance reliability and efficiency.

## Figures and Tables

**Table 1:** Characteristics of participant groups in the assessment experiment.

Characteristic	Voice (n=6)	Text (n=6)	Control (n=6)
Gender			
Male	4	4	3
Female	2	2	3
Age			
40 to 49 years	1	1	0
50 to 59 years	3	4	2
60 to 69 years	2	1	4
Do you use a mobile device?			
Not often	0	0	0
1×/week	0	0	0
3×/week	0	0	1
5×/week	1	0	0
Daily	5	6	5
Prior knowledge of conversational agents			
Knows and has used it	1	2	0
Knows, but never used	0	1	1
Does not know	5	3	5
Self-affirmed level of technological knowledge			
Advanced	0	2	0
High intermediate	0	0	0
Intermediary	1	0	1
Basic	3	2	3
Extremely basic	2	2	2

**Table 2:** Comparative summary of qualitative findings across groups, by aspect.

Aspect	Control (n=6)	Text (n=6)	Voice (n=6)
Overall experience	Positive and easy to handle; described as a valid means of monitoring clinical status.	Interesting and innovative; high perceived utility.	Innovative and easy to learn; some initial confusion that resolved with adaptation.
Difficulties (observed in)	2/6: general app navigation and item deletion.	1/6: limited digital experience.	3/6: diction and learning verbal commands.
Facilitators	Logical block flow, system readability, speed.	Simplicity of design and ease of manual handling.	Clear explanations, easy application, effective data verification.
Suggestions	Simplify item deletion; expand beyond hypertension to additional comorbidities.	Add prompts for medication timing; continued refinement.	Use the technology for broader healthcare initiatives.

**Table 3:** Per-group descriptive statistics and between-group ANOVA results for each UEQ scale, with $\eta^{2}$ effect sizes and Cohen’s benchmark magnitude reported as descriptive aids given the small sample. Mean (SD) [±95% CI] are reported per group as produced by the UEQ analysis tool. ANOVA terms use $df_{between}\;=\;2$ and $df_{within}\;=\;15$.

	Mean (SD) [±95% CI] per Group			Between-Group ANOVA			
Scale	Control (n=6)	Text (n=6)	Voice (n=6)	F	*p*	$\eta^{2}$	Magnitude
Attractiveness	2.37 (0.82) [±1.66]	1.86 (1.09) [±0.87]	2.28 (0.70) [±0.56]	0.53	0.599	0.066	Medium
Perspicuity	1.95 (0.65) [±1.38]	1.42 (1.35) [±1.08]	1.88 (1.01) [±0.81]	0.42	0.665	0.053	Small
Efficiency	2.40 (0.52) [±1.95]	1.88 (1.12) [±0.89]	2.50 (0.73) [±0.58]	0.93	0.416	0.110	Medium
Dependability	2.05 (0.48) [±1.63]	1.46 (1.04) [±0.83]	2.00 (0.85) [±0.68]	0.87	0.439	0.104	Medium
Stimulation	2.30 (0.89) [±1.52]	1.50 (1.16) [±0.93]	2.42 (0.59) [±0.47]	1.76	0.206	0.190	Large
Novelty	2.10 (1.01) [±1.22]	1.54 (1.22) [±0.98]	1.92 (0.96) [±0.77]	0.39	0.684	0.049	Small
Pragmatic Quality	2.13 (0.55) [±1.65]	1.58 (1.17) [±0.94]	2.13 (0.86) [±0.69]	0.87	0.439	0.104	Medium
Hedonic Quality	2.20 (0.95) [±1.37]	1.52 (1.19) [±0.95]	2.17 (0.77) [±0.62]	1.67	0.221	0.182	Large

## Data Availability

The original contributions presented in this study are included in the Supplementary Materials. Further inquiries can be directed to the corresponding authors.

## Supplementary Materials

The following supporting information can be downloaded from https://www.mdpi.com/article/10.3390/healthcare14121641/s1: File S1: standardized task script for the text-based chatbot; File S2: standardized task script for the voice-based agent; File S3: standardized task script for the conventional eProHealth app; File S4: open-ended questionnaire used to capture qualitative feedback; File S5: anonymized raw dataset; File S6: Informed consent form.

## Abbreviations

The following abbreviations are used in this manuscript:

ANOVA	Analysis of Variance
API	Application Programming Interface
AWS	Amazon Web Services
BP	Blood Pressure
CI	Confidence Interval
JSON	JavaScript Object Notation
JWT	JSON Web Token
LGPD	Brazilian General Data Protection Law
NLP	Natural Language Processing
REST	Representational State Transfer
SD	Standard Deviation
UEQ	User Experience Questionnaire
UX	User Experience
