# Supplementary material for: Design, Development, and Evaluation of Multimodal Conversational Agents for Health Data Registration and Monitoring: Framework Proposal and Pilot Exploratory Study
Source: Healthcare (Basel). 2026 Jun 10;14(12):1641. doi: 10.3390/healthcare14121641 (PMC13299244; doi:10.3390/healthcare14121641)
Supplement: Supplementary file 1 [file healthcare-14-01641-s001.zip › File S1 - standardized task script for the text-based chatbot.pdf]

APÊNDICE E – ROTEIRO CHATBOT

UNIVERSIDADE DE PASSO FUNDO
Instituto de Ciências Exatas e Geociências(ICEG)
Programa de Pós-Graduação em Computação Aplicada

ROTEIRO 1

IDENTIFICADOR DO USUÁRIO:__________________ DATA:___/___/_____

A fim de avaliar o produto, por favor siga o seguinte roteiro definido. Em cada etapa, você deve seguir de forma ordenada todos os passos. Após finalizar a etapa, marque que ela já foi realizada e passe para a próxima.

ETAPA 1	**COM O CHATBOT JÁ CONFIGURADO NA TELA INICIAL**<br>1. Escreva "Novo registro" ou selecione o botão "Inserir ".	(___) Já realizei
	2. Selecione o botão "Adicionar Pressão Arterial" ou escreva "Adicionar Pressão Arterial".	(___) Já realizei
	3. Após receber a resposta de retorno do Chatbot, escreva apenas o valor da sua Pressão Sistólica.<br>a. A pressão sistólica é a pressão de saída de sangue do coração, seu registro geralmente é aferido entre 100 mmHg e 160 mmHg.	(___) Já realizei
	4. Após receber a resposta de retorno do Chatbot, escreva apenas o valor da sua Pressão Diastólica.<br>a. A pressão diastólica é a pressão de entrada de sangue do coração, seu registro geralmente é aferido entre 60 mmHg e 90 mmHg.	(___) Já realizei
	5. Após receber a resposta de retorno do Chatbot, clique ou escreva se você tomou um não seu medicamento.	(___) Já realizei
	6. Após receber a resposta de retorno do Chatbot, defina para qual data você quer adicionar este registro, podendo ser uma data no presente ou no passado.	(___) Já realizei
	7. Após receber a resposta de retorno do Chatbot, defina para qual horário você quer adicionar este registro.	(___) Já realizei
	8. Após receber a resposta de retorno do Chatbot, confirme os dados de inserção.	(___) Já realizei

	9. Após receber a resposta de retorno do Chatbot, seu primeiro fluxo estará finalizado.	(___) Já realizei
	10. Clique em voltar ao menu Inicial e passe para a próxima etapa.	(___) Já realizei
ETAPA 2	1. Selecione o botão "Consultar registro" ou escreva "Consultar registro".	(___) Já realizei
	2. Após receber a resposta de retorno do Chatbot, escreva ou selecione o tipo de registro que você quer consultar.<br>a. Você pode consultar um registro de Pressão Arterial, de Sono, de Gordura Corporal, de Batimentos Cardíacos, de Peso e de Cintura.	(___) Já realizei
	3. Após receber a resposta de retorno do Chatbot, a segunda etapa estará finalizada. Clique em voltar ao menu Inicial e passe para a próxima etapa.	(___) Já realizei
ETAPA 3	1. Selecione o botão "Excluir registro" ou escreva "Excluir registro".	(___) Já realizei
	2. Após receber a resposta de retorno do Chatbot, escreva ou selecione o tipo de registro que você quer excluir.<br>a. Você pode excluir um registro de Pressão Arterial, de Sono, de Gordura Corporal, de Batimentos Cardíacos, de Peso e de Cintura.	(___) Já realizei
	3. Após receber a resposta de retorno do Chatbot, confirme os a exclusão do seu último registro.	(___) Já realizei
	4. Após receber a resposta de retorno do Chatbot, seu fluxo estará finalizado.	(___) Já realizei

I
