# Supplementary material for: Design, Development, and Evaluation of Multimodal Conversational Agents for Health Data Registration and Monitoring: Framework Proposal and Pilot Exploratory Study
Source: Healthcare (Basel). 2026 Jun 10;14(12):1641. doi: 10.3390/healthcare14121641 (PMC13299244; doi:10.3390/healthcare14121641)
Supplement: Supplementary file 1 [file healthcare-14-01641-s001.zip › File S2 - standardized task script for the voice-based agent.pdf]

APÊNDICE F – ROTEIRO VOICEBOT

UNIVERSIDADE DE PASSO FUNDO
Instituto de Ciências Exatas e Geociências(ICEG)
Programa de Pós-Graduação em Computação Aplicada

ROTEIRO 2

IDENTIFICADOR DO USUÁRIO:__________________ DATA:___/___/_____

A fim de avaliar o produto, por favor siga o seguinte roteiro definido. Em cada etapa, você deve seguir de forma ordenada todos os passos. Após finalizar a etapa, marque que ela já foi realizada e passe para a próxima.

Observação importante: O dispositivo utilizado escuta sua voz para realizar comandos. Para que ele escute o comando que você deseja realizar, fale "Alexa" OU veja se a cor inferior do dispositivo está azul, caso esteja azul você pode falar que o dispositivo estará escutando.

**ETAPA 1**	**COM O VOICEBOT JÁ CONFIGURADO**<br>1. Diga "Alexa, abrir monitoramento de saúde".	(___) Já realizei
	2. Após receber a resposta de retorno inicial do Voicebot, diga "Inserir registro de pressão" ou diga "Alexa, inserir registro de pressão".	(___) Já realizei
	3. Após receber a resposta de retorno do Voicebot, informe apenas o valor(Número) da sua Pressão Sistólica.<br>a. Ex: Alexa, cento e vinte.<br>b. A pressão sistólica é a pressão de saída de sangue do coração, seu registro geralmente é aferido entre 100 mmHg e 160 mmHg.	(___) Já realizei
	4. Após receber a resposta de retorno do Voicebot, confirme o dado de Pressão Sistólica.	(___) Já realizei
	5. Após receber a resposta de retorno do Voicebot, informe apenas o valor da sua Pressão Diastólica.<br>a. Ex: Alexa, oitenta.<br>b. A pressão diastólica é a pressão de entrada de sangue do coração, seu registro geralmente é aferido entre 60 mmHg e 90 mmHg.	(___) Já realizei
	6. Após receber a resposta de retorno do Voicebot, confirme o dado de Pressão Diastólica.	(___) Já realizei

	7. Após receber a resposta de retorno do Voicebot, informe se você tomou ou não seu medicamento.	(___) Já realizei
	8. Após receber a resposta de retorno do Voicebot, informe para qual data você quer adicionar este registro, podendo ser uma data no presente ou no passado.	(___) Já realizei
	9. Após receber a resposta de retorno do Voicebot, confirme a data de inserção do seu registro.	(___) Já realizei
	10. Após receber a resposta de retorno do Voicebot, informe para qual hora você quer adicionar este registro.	(___) Já realizei
	11. Após receber a resposta de retorno do Voicebot, confirme a hora de inserção do seu registro.	(___) Já realizei
	12. Após receber a resposta de retorno do Voicebot, seu primeiro fluxo estará finalizado.	(___) Já realizei
**ETAPA 2**	1. Diga "Alexa, consultar um registro".	(___) Já realizei
	2. Após receber a resposta de retorno do Voicebot, diga o tipo de registro que você quer consultar.<br>a. Ex: Alexa,consultar registro de Pressão Arterial.<br>b. Você pode consultar um registro de Pressão Arterial, de Sono, de Gordura Corporal, de Batimentos Cardíacos, de Peso e de Cintura.	(___) Já realizei

	3. Após receber a resposta de retorno do Voicebot, a segunda etapa estará finalizada.	(___) Já realizei
E T A P A 3	1. Diga "Alexa, excluir um registro".	(___) Já realizei
	2. Após receber a resposta de retorno do Voicebot, diga o tipo de registro que você quer excluir.<br>a. Ex: Alexa, excluir registro de pressão arterial<br>b. Você pode excluir um registro de Pressão Arterial, de Sono, de Gordura Corporal, de Batimentos Cardíacos, de Peso e de Cintura.	(___) Já realizei
	3. Após receber a resposta de retorno do Voicebot, confirme os a exclusão do seu último registro.	(___) Já realizei
	4. Após receber a resposta de retorno do Voicebot, seu fluxo estará finalizado.	(___) Já realizei
