# Supplementary material for: Design, Development, and Evaluation of Multimodal Conversational Agents for Health Data Registration and Monitoring: Framework Proposal and Pilot Exploratory Study
Source: Healthcare (Basel). 2026 Jun 10;14(12):1641. doi: 10.3390/healthcare14121641 (PMC13299244; doi:10.3390/healthcare14121641)
Supplement: Supplementary file 1 [file healthcare-14-01641-s001.zip › File S3 - standardized task script for the conventional eProHealth app.pdf]

APÊNDICE G – ROTEIRO APLICATIVO

UNIVERSIDADE DE PASSO FUNDO
Instituto de Ciências Exatas e Geociências(ICEG)
Programa de Pós-Graduação em Computação Aplicada

ROTEIRO 3

IDENTIFICADOR DO USUÁRIO:__________________ DATA:___/___/_____

A fim de avaliar o produto, por favor siga o seguinte roteiro definido. Em cada etapa, você deve seguir de forma ordenada todos os passos. Após finalizar a etapa, marque que ela já foi realizada e passe para a próxima.

**ETAPA 1**	**COM O APLICATIVO JÁ CONFIGURADO NA TELA INICIAL**<br>1. Clique no retângulo "Pressão Arterial".	(___) Já realizei
	2. Após a tela de Registros de Pressão Arterial carregar, clique em "+Novo" para adicionar um novo registro.	(___) Já realizei
	3. Após a tela de inserção carregar, defina para qual **data** você quer adicionar este registro, podendo ser uma data no presente ou no passado.	(___) Já realizei
	4. Após, informe apenas a **hora** do registro.	(___) Já realizei
	5. Após, informe apenas o valor de Pressão Sistólica.<br>a. A pressão sistólica é a pressão de saída de sangue do coração, seu registro geralmente é aferido entre 100 mmHg e 160 mmHg.	(___) Já realizei
	6. Após, informe apenas o valor de Pressão Diastólica.	(___) Já realizei
	7. Após, informe apenas o valor de seu Batimento Cardíaco.	(___) Já realizei
	8. Selecione se tomou ou não seu medicamento.	(___) Já realizei
