# Supplementary material for: Design, Development, and Evaluation of Multimodal Conversational Agents for Health Data Registration and Monitoring: Framework Proposal and Pilot Exploratory Study
Source: Healthcare (Basel). 2026 Jun 10;14(12):1641. doi: 10.3390/healthcare14121641 (PMC13299244; doi:10.3390/healthcare14121641)
Supplement: Supplementary file 1 [file healthcare-14-01641-s001.zip › File S4 - open-ended questionnaire used to capture qualitative feedback.pdf]

APÊNDICE D – QUESTIONÁRIO DESCRITIVO

UNIVERSIDADE DE PASSO FUNDO
Instituto de Ciências Exatas e Geociências(ICEG)
Programa de Pós-Graduação em Computação Aplicada

IDENTIFICADOR DO USUÁRIO:____________________ DATA:___/___/_____

QUESTIONÁRIO DESCRITIVO

1- Descreva como foi sua experiência no uso da solução?

____

____

____

____

____

2- Quais as dificuldades encontradas durante o uso da solução?

____

____

____

____

____

3- Quais as facilidades encontradas durante o uso da solução?

____

____

____

____

____

4- Alguma sugestão sobre a solução?

____

____

____

____

____
