# Supplementary material for: Design, Development, and Evaluation of Multimodal Conversational Agents for Health Data Registration and Monitoring: Framework Proposal and Pilot Exploratory Study
Source: Healthcare (Basel). 2026 Jun 10;14(12):1641. doi: 10.3390/healthcare14121641 (PMC13299244; doi:10.3390/healthcare14121641)
Supplement: Supplementary file 1 [file healthcare-14-01641-s001.zip › File S6 - consent-form.pdf]

UNIVERSIDADE DE PASSO FUNDO
Instituto de Ciências Exatas e Geociências(ICEG)
Programa de Pós-Graduação em Computação Aplicada

TERMO DE CONSENTIMENTO LIVRE E ESCLARECIDO (TCLE)

Você está sendo convidado(a) a participar da pesquisa "UM MODELO DE ATENÇÃO HUMANIZADO MULTIMODAL PARA MELHORAR A UX EM APLICATIVO DE SAÚDE DIGITAL", de responsabilidade da pesquisadora Ana Carolina B. De Marchi, docente do Programa de Pós-graduação em Computação Aplicada da Universidade de Passo Fundo.

Esta pesquisa busca identificar se a utilização de *voicebots* pode auxiliar o registro de dados de saúde e, consequentemente, melhorar a experiência do usuário ao interagir com aplicativos de saúde. O objetivo desta pesquisa é propor um modelo de atenção humanizado para melhorar a experiência e as condições de saúde do usuário a partir da multimodalidade e interoperabilidade em aplicativo de saúde digital. Ainda, busca-se mapear possibilidades de interação com o assistente que influenciam a experiência do usuário ao interagir com m-Health.

A sua participação na pesquisa será em uma única sessão, presencial, realizada na Unidade Básica de Saúde, com duração aproximada de 25 minutos, sendo 10 a 20 minutos para a utilização do agente conversacional e o restante para o preenchimento dos instrumentos avaliativos.

Se for identificado algum sinal de desconforto ao responder os instrumentos e/ou durante o uso do aplicativo, o pesquisador compromete-se em orientá-lo(a) e encaminhá-lo(a) para os profissionais especializados na área. Ao participar da pesquisa, você terá os seguintes benefícios: a) a possibilidade de utilizar um aplicativo que auxilia no gerenciamento das condições de saúde; b) dispor de uma solução que estimula a mudança de estilo de vida; e c) obter orientações sobre tecnologias aplicadas à saúde para prevenir e avaliar riscos de doenças e agravos. Você terá a garantia de receber esclarecimentos sobre qualquer dúvida relacionada à pesquisa e poderá ter acesso aos seus dados em qualquer etapa do estudo.

Sua participação nesta pesquisa não é obrigatória e você pode desistir a qualquer momento, retirando seu consentimento. Você não terá qualquer despesa para participar da presente pesquisa e não receberá pagamento pela sua participação no estudo. Caso ocorra eventual dano comprovadamente decorrente da sua participação na pesquisa, você tem o direito de buscar indenização. As suas informações serão armazenadas em computador de acesso restrito aos pesquisadores do estudo e posteriormente destruídas. Os dados relacionados à sua identificação não serão divulgados. A coleta de dados será presencial e apenas a pesquisadora e seu orientando, Mateus K. Roman, terão acesso aos dados.

Caso você tenha dúvidas sobre o comportamento dos pesquisadores ou sobre as mudanças ocorridas na pesquisa que não constam no TCLE, e caso se considere prejudicado (a) na sua dignidade e autonomia, você pode entrar em contato com o (a) pesquisador (a) Ana Carolina B. De Marchi pelo telefone (54) 99975-3130, ou com o Programa de Pós-graduação em Computação Aplicada pelo telefone (54) 3316-8354, ou também pode consultar o Comitê de Ética em Pesquisa da UPF, pelo telefone (54) 3316-8157, no horário das 08h às 12h e das 13h30min às 17h30min, de segunda a sexta-feira. O Comitê está localizado no Campus I da Universidade de Passo Fundo, na BR 285, Bairro São José, Passo Fundo/RS. O Comitê de Ética em Pesquisa exerce papel consultivo e, em especial, educativo, para assegurar a formação continuada dos pesquisadores e promover a discussão dos aspectos éticos das pesquisas em seres humanos na comunidade. Dessa forma, se você concorda em participar da pesquisa como consta nas explicações e orientações acima, coloque seu nome no local indicado abaixo. Desde já, agradecemos a sua colaboração e solicitamos a sua assinatura de autorização neste termo, que será também assinado pelo pesquisador responsável em duas vias, sendo que uma ficará com você e outra com o (a) pesquisador (a).

Passo Fundo, ____ de ____ de _______.

Nome do (a) participante:______________________________

Assinatura: ____

Nome do (a) pesquisador (a): Ana Carolina Bertoletti De Marchi

Assinatura: Ana Carolina B. De Marchi
